# Real World Experience of Chronic Hepatitis C Retreatment with Genotype Specific Regimens in Nonresponders to Previous Interferon-Free Therapy

**DOI:** 10.1155/2019/4029541

**Published:** 2019-03-03

**Authors:** Dorota Zarębska-Michaluk, Iwona Buczyńska, Krzysztof Simon, Magdalena Tudrujek-Zdunek, Ewa Janczewska, Dorota Dybowska, Marek Sitko, Beata Dobracka, Jerzy Jaroszewicz, Paweł Pabjan, Jakub Klapaczyński, Łukasz Laurans, Włodzimierz Mazur, Łukasz Socha, Olga Tronina, Miłosz Parczewski, Robert Flisiak

**Affiliations:** ^1^Department of Infectious Diseases, Voivodship Hospital and Jan Kochanowski University, Kielce, Poland; ^2^Department of Infectious Diseases and Hepatology, Wrocław Medical University, Wrocław, Poland; ^3^Department of Infectious Diseases and Hepatology, Medical University of Lublin, Lublin, Poland; ^4^Department of Basic Medical Sciences, School of Public Health in Bytom, Medical University of Silesia, Katowice, Poland; ^5^Department of Infectious Diseases and Hepatology, Faculty of Medicine, Collegium Medicum Bydgoszcz, Nicolaus Copernicus University Toruń, Toruń, Poland; ^6^Department of Infectious and Tropical Diseases, Jagiellonian University Collegium Medicum, Kraków, Poland; ^7^MED-FIX Medical Center, Wrocław, Poland; ^8^Department of Infectious Diseases, Medical University of Silesia in Katowice, Bytom, Poland; ^9^Department of Internal Medicine and Hepatology, Central Clinical Hospital of the Ministry of Internal Affairs and Administration, Warsaw, Poland; ^10^Multidisciplinary Regional Hospital in Gorzów Wielkopolski, Gorzów Wielkopolski, Poland; ^11^Department of Infectious Diseases, Hepatology, and Liver Transplantation, Pomeranian Medical University, Szczecin, Poland; ^12^Clinical Department of Infectious Diseases, Specialist Hospital in Chorzów, Medical University of Silesia, Katowice, Poland; ^13^Department of Transplantation Medicine, Nephrology, and Internal Diseases, Medical University of Warsaw, Warsaw, Poland; ^14^Department of Infectious, Tropical Diseases and Acquired Immunodeficiency, Pomeranian Medical University, Szczecin, Poland; ^15^Department of Infectious Diseases and Hepatology, Medical University of Białystok, Białystok, Poland

## Abstract

**Background and Aim:**

The development of interferon- (IFN-) free regimens substantially improved efficacy of treatment for HCV, but despite excellent effectiveness the failures still occur. The aim of our study was to evaluate the efficacy of retreatment with genotype specific direct acting antivirals- (DAA-) based regimens in nonresponders to previous IFN-free therapy.

**Materials and Methods:**

Analysed population consisted of 31 nonresponders to IFN-free regimen, which received second IFN-free rescue therapy, selected from 6228 patients included in a national database EpiTer-2.

**Results:**

Age and gender distribution were similar, whereas proportion of genotype 1b was slightly higher and genotype 4 lower in the whole population compared to studied one. Patients included in the study demonstrated much more advanced fibrosis. Primary therapy was discontinued in 12 patients, which were recognized as failures due to nonvirologic reason, whereas virologic reason of therapeutic failure was recognized in 19 patients which completed therapy. Overall sustained virologic response (SVR) rate was 81% and 86% in intent-to-treat (ITT) and modified ITT analysis, respectively (74% and 78% in virologic failures, 92% and 100% in nonvirologic failures). Resistance-associated substitutions (RAS) testing was carried out in 8 patients from the group of completed primary therapy and three of them had potential risk for failure of rescue therapy due to NS5A association, while two of them achieved SVR.

**Conclusions:**

We demonstrated moderate effectiveness of genotype specific rescue therapy in failures due to virologic reason and high in those who discontinued primary therapy. Therefore rescue therapy with genotype specific regimens should be considered always if more potent regimens are not available.

## 1. Introduction

According to recent estimates, chronic infection with hepatitis C virus (HCV) affects approximately 71 million individuals worldwide [1]. The introduction of direct acting antiviral (DAA) therapies significantly improved treatment outcomes. DAA-based options are highly effective with sustained virologic response (SVR) rate exceeding 90% irrespective of liver disease severity and history of previous therapy [2]. Despite excellent effectiveness of all-oral regimens, the failures to eradicate HCV RNA still occur. From 2015 retreatment strategies recommended by European Association for the Study of the Liver (EASL) for nonresponders to prior DAA-containing therapy included four interferon- (IFN-) free options depending on HCV genotype and known resistance profiles of the previously used drugs [3, 4]. Three of them were genotype specific: Sofosbuvir plus Ledipasvir with optional ribavirin (SOF/LDV ± RBV), Ombitasvir/Paritaprevir/ritonavir ± Dasabuvir ± ribavirin (OBV/PTV/r ± DSV ± RBV), and SOF combined with Simeprevir (SMV) or Daclatasvir (DCV). Additionally to these options, new combinations became recommended by EASL guidelines since 2016 for prior DAA nonresponders: Grazoprevir/Elbasvir (GZR/EBR) for infected with genotype 1 or 4 and pangenotypic SOF/Velpatasvir (VEL). Moreover four rescue regimens were recommended for failures of NS5A inhibitor-containing therapy: SOF+ OBV/PTV/r+DSV, SOF+GZR/EBR, and SOF plus SMV or DCV. All listed treatment options should be given with weight-based RBV for 12 weeks in mild or moderate fibrosis and for 24 weeks in extensive fibrosis or cirrhosis [5].

Since EASL recommendations for prior DAA-based nonresponders were supported by few clinical trials including a small number of patients, retreatment policy for this subpopulation had no sufficient background. Moreover it was difficult to apply in real world practice due to delayed registration and reimbursement regulations of novel therapeutic options in numerous countries. There are limited data on the virological outcomes of HCV patients retreated after DAA failure in real world setting. The aim of our study was to evaluate the efficacy of retreatment with genotype specific regimens administered in patients after IFN-free therapy.

## 2. Materials and Methods

We investigated study population consisted of nonresponders to IFN-free regimen, which received second IFN-free rescue therapy. They were selected from the EpiTer-2, an investigator-initiated study, supported by the Polish Association of Epidemiologists and Infectiologists, which included 22 Polish centres involved in diagnosis and treatment of HCV-infected patients. As presented in the previous publications from the EpiTer-2 study data of consecutive patients who started antiviral therapy after 1 July, 2015, and completed before December 2017 were collected retrospectively with a web-based questionnaire [6–8]. Patients were treated in the therapeutic programme reimbursed by the National Health Fund and consented for treatment and medical procedures according to the standard of care and their data were entered retrospectively into the EpiTer-2 database.

Patients were selected for this study based on analysis of previous therapy history. Among 6228 patients included in the EpiTer-2 database 31 patients were previously treated and failed interferon-free, DAA-based genotype specific regimens, and then were retreated again with a genotype specific, interferon-free rescue therapy. Retreatment regimen for particular patient was assigned based on the physician's decision, included in some patients results of RAS testing.

NS3 and NS5A sequencing were performed using previously published methodology with sequence assembly performed using the Recall online tool and verified with Oxford HCV Automated Subtyping Tool and the COMET subtyping tool [9]. RAS were called using 15% threshold and identified using geno2pheno algorithm HCV v.0.92, similarly to the previously published datasets [10].

Since there is no access in analysed period to pangenotypic therapeutic options as well as complex regimens recommended recently by EASL, IFN-free retherapy was possible with genotype specific regimens only. The effectiveness end point was the rate of patients who achieved SVR12, defined as undetectable HCV RNA 12 weeks after treatment termination. Baseline, ontreatment and follow-up data were collected retrospectively by online questionnaire dedicated to EpiTer-2 database. Effectiveness of therapy was calculated according to intent-to-treat (ITT) and modified ITT (mITT) analysis that excluded patients lost to follow-up.

## 3. Results

Age and gender distribution in this group (Table 1) were similar to observed in the whole population of patients included into EpiTer-2 database, which were 54 ± 32 years of age and 52%/48%, respectively, whereas proportion of genotype 1b was slightly higher (83%) and genotype 4 lower (4%) in the whole population compared to studied one (Table 1). Patients included in the study demonstrated also much more advanced fibrosis, because proportion of cirrhotics in all EpiTer-2 population was 33% compared to 71% (22/31) in the study group.

The most frequent primary regimen administered to 15 patients (48%) of the studied population was OBV/PTV/r+DSV ± RBV, which was discontinued due to adverse events in 11 patients (Table 1). Altogether 12 patients received primary therapy for 1 to 4 weeks (one more patient received ASV+DCV by mistake and was switched to OBV/PTV/r+RBV). To differentiate patients who completed primary therapy as scheduled from those who stopped it due to different reason sometimes even after very short treatment period further analysis was carried out in two groups of those who completed or discontinued treatment.

**Figure 1:**
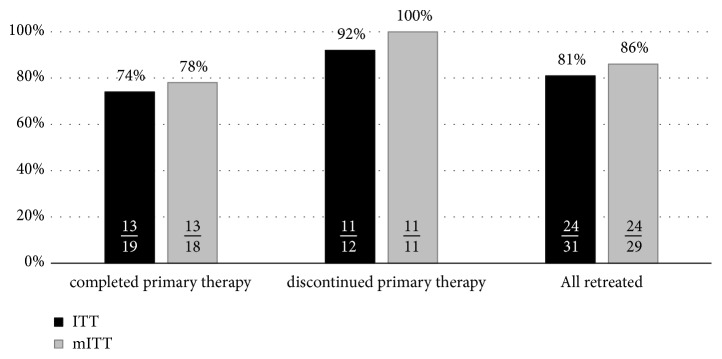
Efficacy of retreatment according to intent-to-treat (ITT) or modified ITT (mITT) analysis in all retreated and based on completed or discontinued primary therapy; mITT analysis was carried out without lost to follow-up patients.

Overall sustained virologic response rate of rescue retreatment in all nonresponders to DAA-based primary therapy (ITT) was 81% (Figure 1). SVR rate calculated after exclusion of two patients lost to follow-up (mITT) reached 86%. All patients with available follow-up data, who discontinued primary therapy, responded to rescue therapy. The SVR rate among those who completed but failed primary therapy was 74% and 78% for ITT and mITT, respectively (Figure 1). Efficacy of particular rescue regimens administered in both groups of completed and discontinued patients were similar (Tables 2 and 3).

RAS testing was carried out in 11 patients (35%), including 8 (42%) from the group of patients which completed primary therapy (Table 4). Presence of RAS was demonstrated in 4 patients. In 3 of them NS5A RAS were found, which might have negatively influenced the efficacy of the rescue therapy (patient 23, 25, and 31), but finally two of these patients achieved SVR. As shown in Tables 4 and 5 presence of RAS as a reason of rescue therapy failure was documented in one patient only (patient 23). In two others (patient 16, 22) RAS testing was not performed, but resistance to NS5A inhibitors should be considered as a possible reason of the failure (Table 5). Improper regimen selection due to genotyping error was the most likely failure reason of both primary and rescue therapy in patient 1.

## 4. Discussion

The recent development of interferon-free all-oral regimens substantially improved efficacy of antiviral treatment for HCV, with cure rate close to 100% in genotype 1b and other genotypes except genotype 3 [6, 7]. Despite the excellent outcome, there is still a group of DAA nonresponders, for which decision about retreatment regimen is a challenge. In our real world study we investigated efficacy of IFN-free retherapy administered to 31 patients who did not achieve SVR with DAA-based regimens. Data on retreated patients with a prior failure to DAA options, particularly NS5A-containing, were limited and optimal regimen was undetermined. Only few clinical trials with a small number of patients supported EASL retreatment recommendations, which were mostly based on indirect evidence [3, 11].

Majority of our DAA nonresponders had advanced liver fibrosis or cirrhosis and due to risk of life-threatening complications retreatment was considered as rescue strategy. They received genotype specific regimens because of unavailability of pangenotypic options at the time of decision. Since one-third of analysed cohort discontinued primary DAA therapy course due to safety reason and only remaining two-thirds were “true” nonresponders we decided to perform separate analysis of these two subgroups, finding reasonable lower efficacy in patients who failed primary course due to virologic reason.

Overall, SVR rate for whole study population was 81% and 86% patients with available follow-up data, respectively, but it was lower (74% and 78%, respectively) in those who completed primary DAA therapy and can be recognized as virologic nonresponders. Our efficacy results are comparable to outcome reported by Lawitz et al. (73%) and lower than achieved by Wilson et al. (91%) [12, 13]. However, in contrast to our study both these trials concerned noncirrhotics failed SOF/LDV and retreated with the longer course of the same regimen. Cooper et al. documented 89% SVR rate, but this study was carried out in HIV/HCV population including cirrhotics, which were retreated with SOF/LDV+RBV after SOF/LDV failure [14]. Similar efficacy (87%) was achieved by Suda et al. in SOF/LDV+RBV retherapy after failure of ASV+DCV regimen used in noncirrhotics [15]. All these studies included NS5A-experienced populations, while for NS5A-naïve cohorts, containing cirrhotic patients, SVR reported for SOF/LDV ± RBV retreatment after prior SOF+RBV or SOF+SMV ± RBV failure, ranged from 88% to 100% [16–21]. Simple comparison of SVR rates seems to be difficult and unreliable due to heterogeneity of populations included in all these studies, in terms of both patient's characteristics and antiviral regimens.

In our analysis all four nonresponders to rescue DAA therapy were NS5A-experienced; in two cases testing for baseline resistance-associated substitutions within NS5A gene was performed and in one case presence of RAS was proven. It is possible that another two NS5A-experienced patients failed NS5A-containing rescue therapy due to resistance, but baseline tests were not performed. According to EASL guidelines utility of HCV resistance testing prior to DAA retherapy in patients who failed previous IFN-free regimen is unknown, but physicians who have access to test analysing HCV resistance to NS5A inhibitors can use these results to select the optimal retreatment strategy [3, 5, 11]. Although presence of resistance-associated substitutions to DAA might impair viral response, still majority of patients with RAS will most likely be cured from HCV infection that was reported previously and supported by presented data [22–29]. In analysed cohort, three nonresponders with documented selection of NS5A substitutions responded to rescue DAA regimen, which is in line with literature data [13–15, 29].

The new DAA options, including pangenotypic drugs, very potent and with a high genetic barrier to resistance, such as velpatasvir, voxilaprevir, glecaprevir, pibrentasvir, have been approved and several are in advanced clinical trials [30–33]. Novel combinations of these drugs may be an effective salvage strategy in patients with previous DAA treatment failure and they can reduce or even exclude need of RAS testing shortly [34]. Presented data of rescue with genotype specific therapy will be useful for comparison with future therapeutic options based on more potent pangenotypic combinations. High rate of difficult-to-treat patients adds to the importance of this real world study.

The major limitation of the study was relatively small number of included patients, particularly those with nonresponse related to virologic reason, which was caused by waiting for more potent regimens. However, it must be pointed out that sample size of study cohort does not differ materially from those analysed in cited trials [12–21]. Another weakness was that one-third of studied population was tested for RAS only.

Concluding, we demonstrated moderate effectiveness of genotype specific rescue therapy in failures due to virologic reason and high effectiveness in those who early discontinued primary therapy. Therefore rescue therapy with genotype specific regimens should be considered always if more potent regimens are not available. RAS testing does not seem to be essential but may be helpful for therapy selection after the first failure of DAA-based therapy.

## Figures and Tables

**Table 1:** Characteristics of nonresponders to interferon-free therapy retreated with genotype specific DAA regimens, compared to the whole population of patients included in the EpiTer-2 database.

Parameter	Studied population
	n=31
females/males, n (%)	16/15 (52%/48%)

Age [years], mean ± SD; min-max	54 ± 11; 23-77

HCV genotype, n (%)	
1b	23 (74%)
3	3 (10%)
4	5 (16%)

Fibrosis, n (%)	
F0	1 (3%)
F1	3 (10%)
F2	3 (10%)
F3	2 (6%)
F4	22 (71%)

Primary regimen, on schedule/discontinued, n (%)	19/12 (61%/39%)
OBV/PTV/r+DSV ± RBV, on schedule	4 (13%)
OBV/PTV/r+DSV ± RBV, discontinued	11 (35%)
LDV/SOF ± RBV, on schedule	7 (23%)
LDV/SOF ± RBV, discontinued	0
ASV+DCV, on schedule	3 (10%)
ASV+DCV, discontinued	1 (3%)
SOF+RBV, on schedule	3 (10%)
SOF+RBV, discontinued	0
Other SOF based†, on schedule	2 (6%)
Other SOF based†, discontinued	0

History of primary regimen failure, n (%)	
Discontinued due to adverse events	11 (35%)
Relapse	10 (32%)
Non-response	9 (29%)
Other‡	1 (3%)

Period between primary and rescue therapy [months], mean ± SD; min-max	9.0 ± 5.0; 0-18

†SOF+SMV+RBV and SOF+DCV+RBV.

‡improper primary regimen discontinued after 4 weeks.

DAA: direct acting antivirals; SD: standard deviation; HCV: hepatitis C virus; F: fibrosis; OBV: ombitasvir; PTV: paritaprevir; DSV: dasabuvir; RBV: ribavirin; LDV: ledipasvir; SOF: sofosbuvir; ASV: asunaprevir; DCV: daclatasvir; SMV: simeprevir.

**Table 2:** Efficacy total, by primary regimen including on schedule/discont, by rescue regimen, by GT, and by fibrosis.

	ITT, n (%)		mITT, n (%)	
	completed primary therapy	discontinued primary therapy	completed primary therapy	discontinued primary therapy
by primary regimen				
OBV/PTV/r+DSV ± RBV	3/4 (75%)	10/11 (91%)	3/4 (75%)	10/10 (100%)
LDV/SOF ± RBV	4/7 (57%)	0	4/6 (67%)	0
other SOF based	5/5 (100%)	0	5/5 (100%)	0
ASV+DCV	2/3 (67%)	1/1 (100%)	2/3 (67%)	1/1 (100%)

by rescue regimen				
OBV/PTV/r ± DSV ± RBV	3/4 (75%)	1/1 (100%)	3/4 (75%)	1/1 (100%)
LDV/SOF ± RBV	6/8 (75%)	10/11 (91%)	6/8 (75%)	10/10 (100%)
GZR/EBR	2/3 (67%)	0	2/2 (100%)	0
SOF+DCV ± RBV	2/3 (67%)	0	2/3 (67%)	0

by genotype				
1b	9/12 (75%)	10/11 (91%)	9/12 (75%)	10/10 (100%)
3	2/3 (67%)	0	2/3 (67%)	0
4	3/4 (75%)	1/1 (100%)	3/3 (100%)	1/1 (100%)

by fibrosis				
non-cirrhotics	6/6 (100%)	3/3 (100%)	6/6 (100%)	3/3 (100%)
cirrhotics	8/13 (62%)	8/9 (89%)	8/12 (67%)	8/8 (100%)

GT: genotype; ITT: intent-to-treat; mITT: modified intent-to-treat; OBV: ombitasvir; PTV: paritaprevir; DSV: dasabuvir; RBV: ribavirin; LDV: ledipasvir; SOF: sofosbuvir; ASV: asunaprevir; DCV: daclatasvir; GZR: grazoprevir; EBR: elbasvir.

**Table 3:** Individual efficacy data depending on HCV genotype, hepatic fibrosis, and primary and rescue regimen.

Patient	Fibrosis	GT	Primary regimen	Response to primary reg.	Months between therapies	Rescue regimen	Response to rescue reg.
1	4	3	OBV/PTV/r+DSV+RBV	REL	6	LDV/SOF+RBV, 12wks	NR
2	4	1B	OBV/PTV/r+DSV+RBV	DSC, 1wk	1	LDV/SOF+RBV, 12wks	SVR
3	4	1B	OBV/PTV/r+DSV+RBV	DSC, 2wks	11	LDV/SOF, 12wks	LFU
4	4	1B	OBV/PTV/r+DSV+RBV	DSC, 3wks	11	LDV/SOF, 24wks	SVR
5	1	1B	OBV/PTV/r+DSV	DSC, 4 wks	5	LDV/SOF, 12wks	SVR
6	4	1B	OBV/PTV/r+DSV+RBV	DSC, 4 wks	2	LDV/SOF, 24wks	SVR
7	1	1B	OBV/PTV/r+DSV	DSC, 1 wk	2	LDV/SOF, 12wks	SVR
8	4	1B	OBV/PTV/r+DSV+RBV	DSC, 2wks	17	LDV/SOF, 12wks	SVR
9	4	1B	OBV/PTV/r+DSV+RBV	DSC, 3wks	10	LDV/SOF+RBV, 12wks	SVR
10	4	1B	OBV/PTV/r+DSV+RBV	DSC, 4wks	9	LDV/SOF, 24wks	SVR
11	3	1B	OBV/PTV/r+DSV	NR	12	LDV/SOF+RBV, 12wks	SVR
12	1	1B	OBV/PTV/r+DSV	DSC, 1 day	7	LDV/SOF, 8wks	SVR
13	4	3	SOF+RBV	REL	3.5	SOF+DCV, 12wks → SOF+RBV, 12wks	SVR
14	2	4	SOF+RBV	NR	6	GZR/EBR, 12wks	SVR
15	4	3	SOF+RBV	REL	8	SOF+DCV+RBV 24wks	SVR
16	4	1B	LDV/SOF+RBV	REL	9	LDV/SOF+RBV, 24wks	NR
17	4	4	LDV/SOF+RBV	REL	8	GZR/EBR, 12wks	LFU
18	3	4	LDV/SOF	NR	16	OBV/PTV/r+RBV, 24wks	SVR
19	2	1B	LDV/SOF	REL	16	OBV/PTV/r+DSV, 12wks	SVR
20	0	4	LDV/SOF	REL	7	GZR/EBR+RBV, 16wks	SVR
21	4	1B	LDV/SOF+RBV	NR	17	LDV/SOF+RBV, 24wks	SVR
22	4	1B	LDV/SOF+RBV	NR	14	SOF+DCV+RBV 24wks	NR
23	4	1B	ASV+DCV	NR	4	OBV/PTV/r+DSV+RBV, 12wks	NR
24	4	4	ASV+DCV	UKN	0	OBV/PTV/r+RBV, 24wks	SVR
25	4	1B	ASV+DCV	NR	12	LDV/SOF+RBV, 24wks	SVR
26	4	1B	SMV+SOF+RBV	REL	13	LDV/SOF+RBV, 24wks	SVR
27	4	1B	SOF+DCV+RBV	NR	4	GZR/EBR, 12wks	SVR
28	4	1B	OBV/PTV/r+DSV+RBV	DSC, 4wks	12	LDV/SOF, 24wks	SVR
29	4	1B	OBV/PTV/r+DSV+RBV	REL	12	LDV/SOF, 24wks	SVR
30	4	1B	OBV/PTV/r+DSV+RBV	REL	7	LDV/SOF, 24wks	SVR
31	2	1B	ASV+DCV	NR	18	OBV/PTV/r+DSV+RBV, 12wks	SVR

GT: genotype; OBV: ombitasvir; PTV: paritaprevir; DSV: dasabuvir; RBV: ribavirin; LDV: ledipasvir; SOF: sofosbuvir; ASV: asunaprevir; DCV: daclatasvir; GZR: grazoprevir; EBR: elbasvir; REL: relapse; DSC: discontinued; NR: nonresponder, UKN: unknown; LFU: lost to follow-up.

**Table 4:** Patients with known RAS testing.

Patient	Primary therapy	RAS testing	Secondary therapy	SVR
1	OBV/PTV/r+DSV+RBV 12 weeks	not detected	LDV/SOF+RBV 12 weeks	NO

5	OBV/PTV/r+DSV 4 weeks	not detected	LDV/SOF 12 weeks	YES

7	OBV/PTV/r+DSV 1 week	not detected	LDV/SOF 12 weeks	YES

15	SOF+RBV 24 weeks	not detected	SOF+DCV 12 weeks →SOF+RBV 12 weeks	YES

20	LDV/SOF 8 weeks	not detected	GZR/EBR+RBV 16 weeks	YES

23	ASV+DCV 24 weeks	NS5A: L31V, Y93H NS3: S122R, D168E	OBV/PTV/r+DSV+RBV 12 weeks	NO

25	ASV+DCV 24 weeks	NS5A - Y93Y/H	LDV/SOF+RBV 24 weeks	YES

28	OBV/PTV/r+DSV+RBV 4 weeks	not detected	LDV/SOF 24 weeks	YES

29	OBV/PTV/r+DSV+RBV 12 weeks	not detected	LDV/SOF 24 weeks	YES

30	OBV/PTV/r+DSV+RBV 12 weeks	NS3-170I	LDV/SOF 24 weeks	YES

31	ASV+DCV 24 weeks	NS5A: 31V, 93H	OBV/PTV/r+DSV+RBV 12 weeks	YES

RAS: resistance associated substitution; OBV: ombitasvir; PTV: paritaprevir; DSV: dasabuvir; RBV: ribavirin; LDV: ledipasvir; SOF: sofosbuvir; ASV: asunaprevir; DCV: daclatasvir.

**Table 5:** Possible reason of nonresponse to rescue therapy.

Patient	GT	F	Primary therapy	Out come	Secondary therapy	ETR	Months between therapies	RAS	Comments
1	3	4	OBV/PTV/r +DSV+RBV 12 weeks	REL	LDV/SOF +RBV 12 weeks	YES	6	after 2^nd^ therapy: Not detected	wrong regimens - initially diagnosed as G1b;

16	1B	4	LDV/SOF +RBV 12 weeks	REL	LDV/SOF +RBV 24 weeks	YES	9	Not done	retreatment with the same regimen - possible RAS to NS5A

22	1B	4	LDV/SOF +RBV 12 weeks	NR	SOF+DCV +RBV 24 weeks	NO	14	Not done	unknown non-response reason - possible RAS to NS5A

23	1B	4	ASV+DCV 24 weeks	NR	OBV/PTV/r +DSV+RBV 12 weeks	NO	4	Before 2^nd^ therapy: NS3: S122R, D168E; NS5A: L31V, Y93H	RAS to NS3 and NS5A

GT: genotype; F: fibrosis; ETR: end of treatment; RAS: resistance associated substitution; NS3: nonstructural protein 3; NS5A: nonstructural protein 5A; NR: nonresponder; REL: relapse; OBV: ombitasvir; PTV: paritaprevir; DSV: dasabuvir; RBV: ribavirin; LDV: ledipasvir; SOF: sofosbuvir; ASV: asunaprevir; DCV: daclatasvir; SMV: simeprevir.

## Data Availability

Patients data included in manuscript are available from the corresponding author upon request.
